# Asymptomatic surveillance testing for COVID-19 in health care professional students: lessons learned from a low prevalence setting

**DOI:** 10.1186/s13223-023-00769-4

**Published:** 2023-03-29

**Authors:** Alyssa G. Burrows, Sophia Linton, Jenny Thiele, Prameet M. Sheth, Gerald A. Evans, Stephen Archer, Katharine M. Doliszny, Marcia Finlayson, Leslie Flynn, Yun Huang, Azim Kasmani, T. Hugh Guan, Allison Maier, Adrienne Hansen-Taugher, Kieran Moore, Anthony Sanfilippo, Erna Snelgrove-Clarke, Dean A. Tripp, David M. C. Walker, Stephen Vanner, Anne K. Ellis

**Affiliations:** 1 Department of Medicine, Queen’s University, Kingston, ON Canada; 2 Allergy Research Unit, Watkins 1D, Kingston Health Sciences Centre-KGH Site, 76 Stuart, Kingston, ON K7L2V7 Canada; 3 Department of Biomedical and Molecular Sciences, Queen’s University, Kingston, ON Canada; 4 Gastrointestinal Disease Research Unit (GIDRU), Faculty of Health Science, Queen’s University, Kingston, ON Canada; 5 Pathology and Molecular Medicine, Queen’s University, Kingston, ON Canada; 6 Queen’s CardioPulmonary Unit, Queen’s University, Kingston, ON Canada; 7 School of Rehabilitation Therapy, Queen’s University, Kingston, ON Canada; 8 Faculty of Health Sciences, Queen’s University, Kingston, ON Canada; 9 Kingston, Frontenac, Lennox & Addington (KFL&A) Public Health, Kingston, ON Canada; 10 Department of Family Medicine, Queen’s University, Kingston, ON Canada; 11 Faculty of Health Sciences School of Nursing, Queen’s University, Kingston, ON Canada; 12 Department of Psychology, Anesthesia, Urology, Queen’s University, Kingston, ON Canada; 13 Department of Emergency Medicine, Queen’s University, Kingston, ON Canada; 14 School of Policy Studies, Queen’s University, Kingston, ON Canada

**Keywords:** COVID-19, Infectious disease, Asymptomatic testing, RT-PCR, Health care professional students, Young adults, Respiratory syncytial virus, Influenza a&b, Screening questionnaire

## Abstract

The novel coronavirus disease of 2019 (COVID-19) pandemic has severely impacted the training of health care professional students because of concerns of potential asymptomatic transmission to colleagues and vulnerable patients. From May 27th, 2020, to June 23rd 2021; at a time when B.1.1.7 (alpha) and B.1.617.2 (delta) were the dominant circulating variants, PCR testing was conducted on 1,237 nasopharyngeal swabs collected from 454 asymptomatic health care professional students as they returned to their studies from across Canada to Kingston, ON, a low prevalence area during that period for COVID-19. Despite 46.7% of COVID-19 infections occurring in the 18–29 age group in Kingston, severe-acute-respiratory coronavirus-2 was not detected in any of the samples suggesting that negligible asymptomatic infection occurred in this group and that PCR testing in this setting may not be warranted as a screening tool.

## Introduction

There is strong evidence that severe-acute-respiratory coronavirus-2 (SARS-CoV-2), the virus that causes novel coronavirus disease of 2019 (COVID-19), can be transmitted from asymptomatic and pre-symptomatic individuals [1–3]. Understanding the prevalence of asymptomatic COVID-19 transmission in areas of varying prevalence is critical for implementing measures to mitigate transmission [1]. Nosocomial-acquired infections are a risk for patients and health care providers. A study conducted on health-care workers from a London United Kingdom hospital between March and April 2020 found that 1.1% to 7.1% of participants tested had an asymptomatic SARS-CoV-2 infection and 45% of participants tested had SARS-CoV-2 antibodies in their serum [4, 5]. At the outset of the pandemic in early 2020, there was considerable concern that students, especially those travelling from high COVID-19 prevalence areas, could inadvertently infect colleagues, faculty and patients. Further of the outbreaks reported in Ontario between April 1st 2020 and March 31st, 2021, 12% of cases were attributed to workplace outbreaks with healthcare and educational services being two of the five industries with the highest rates of outbreaks [6]. To our knowledge, asymptomatic SARS-CoV-2 infection data has not yet been reported in the Canadian health care trainee population. COVID-19 severely impacted health care education and the loss of training opportunities had to be balanced with the learners’ and community’s safety [7–10].

Further COVID-19 critically impacted the Canadian post-secondary sector, a survey conducted by Statistics Canada found that of 100,000 post secondary students 35% reported delayed or cancelled work placements and 26% reported that some of their courses were postponed or cancelled [11]. It has been estimated that Canadian universities could lose between $438 million (1.0%) to $2.5 billion (-5.7%) of projected revenue in 2020/2021, to combat these loses the Ontario government has provided $106.4 million to publicly assisted universities and colleges [12, 13].

## Methods

This prospective observational study aimed to identify asymptomatic carriage of SARS-CoV-2 in this occupational group who had in-person educational components, often in a clinical outpatient and inpatient hospital settings, leading to frequent contact with patients, faculty and peers. The Queen’s University Health Sciences Research Ethics Board approved this study. Prospective participants were invited by their schools through email and virtual information sessions. Interested participants contacted the study team, who gained the participant’s verbal consent prior to their enrollment in the study. Queen’s FHS was amongst the first post-secondary schools in Canada to allow for in-person health care professional training during the outbreak of the pandemic in 2020 and many students travelled from their homes chiefly from across Canada to campus, including many from high prevalence areas such as the Greater Toronto Area. Before entry into the study site, participants were contacted by phone one day prior to their visit to complete the Kingston Health Sciences (KHSC) Infection, Prevention & Control COVID-19 screening questionnaires with the research team. Participants were screened on the day of their study visit using the same COVID-19 screening questionnaire and again by member of our study team before entering the study site. Participants were not allowed to enter the study site, as per KHSC’s Infection, Prevention & Control COVID-19 measures if they had symptoms of COVID-19, recent travel to restricted areas, or had been in contact with a suspected or confirmed case of COVID-19. Ten participants were declined entry to the study site for failing KHSC’s COVID-19 screening questions. These participants had their appointments rescheduled and were not excluded from the study. Participants completed one to five visits, spaced a minimum of 2 weeks apart (Visit 1; May 2020 to March 2021 (n = 452), Visit 2; July 2020 to June 2021 (n = 406), Visit 3; August 2020 to June 2021 (n = 266), Visit 4; December 2020 to June 2021 (n = 124); Visit 5; January 2021 to March 2021 (n = 4)). The average number of samples collected from participants was between 3 and 4 samples.

### Questionnaires

At each visit participants completed a survey which was administered on paper and entered into REDCap from May 27th, 2020 to August 13th, 2020. From August 25th, 2020 onward the survey was completed online through Qualtrics^XM^. This questionnaire included demographic, geographic, COVID-19 symptoms, physical health, behavioural, mental health, attitudes towards COVID-19, COVID-19 testing (outside study), Influenza and COVID-19 vaccination status. Behavioural questions included questions about mask use, hand hygiene, supplement intake, physical distancing practices in public, school, and at home, contact with household members with COVID-19 and potential exposure to community members (e.g. grocery shopping).

#### Asymptomatic SARS-CoV-2 nasopharyngeal testing

From May 27th, 2020 to June 23rd, 2021, 1,237 nasopharyngeal (NP) swabs were collected from the posterior wall of the nasopharynx placed in 3 mL of viral media and processed by the KHSC-Kingston General Hospital Clinical Microbiology Laboratory using reverse transcriptions-polymerase chain reaction (RT-PCR) that detected the E-gene and the 5′UTR regions of SARS-CoV-2. All samples were assessed for SARS-CoV-2. Samples taken between January 12th and March 9th, 2021 were also tested or influenza A & B 16.2% (n = 201) and human orthopneumovirus (HOPV) 16.2% (n = 201) which is the species of viruses that includes several clades of respiratory syncytial viruses [14].

#### Blood collection

Blood specimens were collected in four 4 mL SST tubes, centrifuged to extract serum and stored at − 80 °C for batch analysis at the KHSC-KGH site Core Chemistry Laboratory. Blood specimens were evaluated using an FDA approved, clinically validated, serologic chromogenic immunoassay (CMIA) assay on the Abbott ARCHITECT i2000SR system that is available at KHSC to detect IgG antibodies to the SARS-CoV-2 Nucleocapsid, indicating prior SARS-CoV-2 infection.

## Results

### Demographics

Four hundred and fifty-four students from Queen’s University Faculty of Health Sciences (FHS) in the Medicine 63.4% (n = 288), Nursing 22.7% (n = 103), Rehabilitation (i.e. occupational therapy, physiotherapy or rehabilitation science) 6.4% (n = 29), and FHS Graduate studies programs 6.4% (n = 29) participated in this study. The age range for our study was 18 to 52 years old (mean age 24.7 years), 69% of the participants identified as female, 30% male, > 1% unknown, non-binary, or preferred not to disclose.

### SARS-CoV-2 RT-PCR

**Fig. 1:**
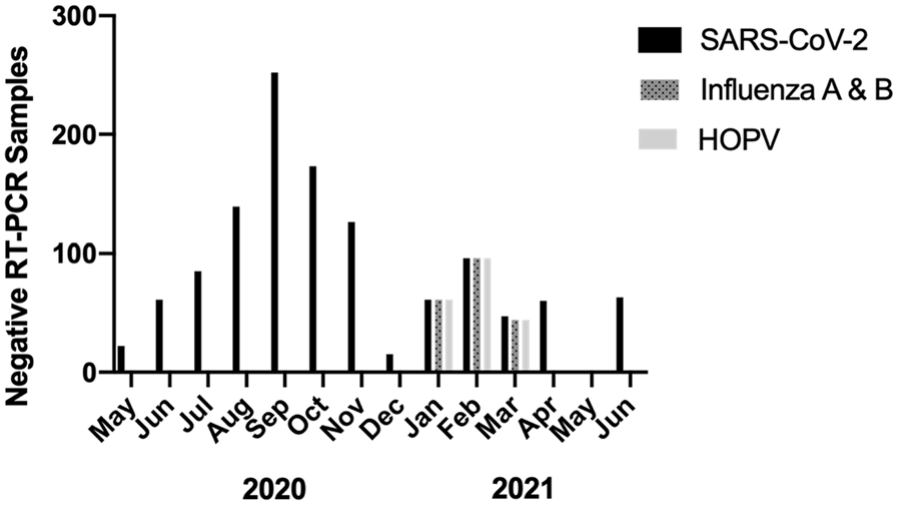
RT-PCR NP samples did not detect SARS-CoV-2 (n = 1200), Influenza A & B (n = 201) and HOPV (n = 201) from asymptomatic health care professional students (n = 457) from Kingston, ON. *HOPV* human Orthopneumovirus, *NP* nasopharyngeal, *RT-PCR* reverse transcriptions-polymerase chain reaction, *SARS-CoV-2* Severe-Acute-Respiratory Coronavirus-2

Of the 1,237 NP swabs, no positive SARS-COV-2 positive samples were found in our asymptomatic health care professional students (Fig. 1). Concurrently, samples collected between January 12th, 2021 and March 9th, 2021, underwent testing for influenza A & B and HOPV. Similarly, no positive samples were detected by RT-PCR (Fig. 1).

### Geographical data

At visit 1 (n = 454 completed questionnaires), 2.86% (n = 13) of participants reported living in a Queen’s residence building, 20.5% (n = 93) reported living alone, 58.6% (n = 266) reported living with non-relatives, 20.0% (n = 91) reported living with relatives. Between September 1st 2019 and March 13th 2020, 65.1% (n = 296) participants reported Kingston as their most recent place of residence, 22.5% (n = 102) reported other cities within Ontario with 13.8% (n = 63) within the Greater Toronto Area, 45 reported a province outside Ontario, 6 reported primary residence outside of Canada. When asked if they moved to a different address from March 14th 2020 to the date of their survey 63.4% (n = 288) responded yes, 10.7% (n = 31) reported living within Kingston, 74.3% (n = 214) report living within Ontario with 40.3% (n = 116) reporting living within the GTA, 23.9% (n = 69) reported living other Canadian provinces and Territories outside Ontario, and 5 reported living in a country outside of Canada. For travel at the screening visited 34.4% (n = 156) participants reported travel between September 1st, 2019 and March 13th, 2020 while 3.3% (n = 15) reported traveling between March 14th 2020 to date of their survey.

### Vaccination

Pfizer and Moderna mRNA COVID-19 vaccines were approved by Health Canada in December 2020. Between January 11th 2021 and June 23rd 2021, 374 visits were completed, 172 vaccines were received, 135 received one dose and 37 received two doses. The days between first and second doses ranged from 21 to 107 days with an average of 56.2 days between the first and second doses. All vaccinations were mRNA vaccines, predominately Pfizer.

### COVID-19 infections

Serum samples (n = 1229) were collected prospectively from Queen’s University Faculty of Health Sciences students (n = 454) to determine seroprevalence to SARS-CoV-2. 0.44% (n = 5 samples, collected from two participants) of the samples met the positive threshold cut-off (> 1.40 RLU) for IgG to the nucleocapsid on the clinically validated Abbott assay. Throughout the duration of the study, two participants reported tested positive for SARS-CoV-2 in their questionnaire.

## Discussion

**Fig. 2:**
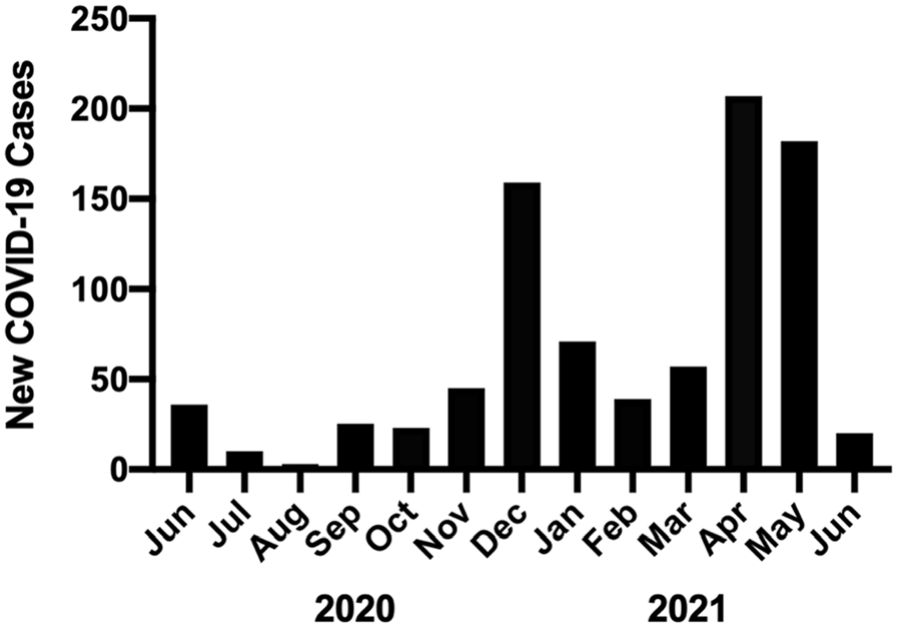
Kingston, Frontenac, Lennox & Addington COVID-19 case load between June 15th, 2020, to June 23rd, 2021. Generated from KFL&A public health data

During the study's time frame, the Kingston, Frontenac, Lennox & Addington (KFL&A) public health region reported 1,494 confirmed cases of COVID-19 (Fig. 2). The test positivity rate ranged between 0.0% to 2.01% (May 8th, 2021). 35.91% and 45.7% of cases were identified as B.1.1.7 (alpha) B.617.2 (delta), respectively. KFL&A was considered a low prevalence area for COVID-19 throughout the first, second and third wave. However, the highest prevalence of COVID-19 cases occurred in the 18- to 29-year-old age category (697 cases; 46.7%) in the highest population density area, including the downtown core in which many students live [15]. Between August 31st, 2020 to June 24th, 2021, Queen’s University reported a total of 317 COVID-19 infections within the student population. Off campus students accounted for 88% (n = 279) while students in residences accounted for 12% (n = 38). Overall, SARS-CoV-2 infections in the Queen’s University population accounted for 20.4% of the KFL&A’s public health region’s SARS-CoV-2 infections (n = 1557) during the same period [16].

The KFL&A public health team recorded two outbreaks in Queen’s University residences which met the definition of a congregate setting from March 11th, 2021 to April 1st, 2021 (n = 17) and April 24th, 2021 to May 18th, 2021 (n = 18) (data provided by KFL&A Public Health). The news media reported on 4 infections in September, 54 cases being associated with house parties in December 2020 and 36 cases in April 2021 within the Queen’s University student population [17–19]. A concern upon initiation of this study was this occupational groups interaction with patients in long term care and clinical settings increasing their risk for COVID-19. Outbreaks in term care facilities and retirement homes between October 2020 and June 2021 were limited to 10 outbreaks comprised of 13 cases total. Additionally, there were no outbreaks in KHSC hospitals or Providence Care throughout the duration of this study (data provided by KFL&A Public Health).

Several post-secondary institutions in the United States have published their COVID-19 measures and outcomes in allowing students to engage in in person learning. Indiana University with 12,000 students (8000 undergraduates 85% of whom live on campus) returned to in-person learning in August 2020. Within two weeks they experienced an outbreak of 371 cases mainly from students living off campus and pivoted to remote learning and 2-weeks of isolation before returning to in-person learning. Similar to our study, increased testing, tracing, and isolation measures allowed the University of Indiana to return to in-person learning [20]. The Public University Campus in Washington State enrolled 16, 476 individuals and performed 29, 783 SARS-CoV-2 test throughout fall 2020, they detected 236 infections representing 0.79% of their swabs. Seventy-five percent of positive cases reported at least 1 of the following: symptoms (60.8%), exposure (34.7%), or high-risk behaviour (21.5%) [21]. In comparison with our smaller health care professional student cohort we did not detect SARS-CoV-2 in the 1200 NP samples collected, this further demonstrated the utility of public health screening questionnaires as the aforementioned study reports that many symptoms and exposures were risk factors in testing positive.

Holiday breaks such as spring break warranted additional caution. Specifically, the Chicago Department of Public Health identified 158 cases among undergraduate students in the city’s university between March 15th, and May 3rd 2021, of infected students 63.6% reported recent travel outside Chicago for spring break and 40.7% reported indoor social exposure [22].

Some participants engaged in intra-provincial, national, and international travel throughout the study for personal or training-related purposes (i.e., residency placements in other cities), which may have increased their risks of contracting SARS-CoV-2 however the number of SARS-CoV-2 infections identified through questionnaires and serology testing in our study was too low to assess these risk factors.

Despite these potential increased risks to COVID-19 exposure, our study revealed that asymptomatic RT-PCR NP testing of a higher risk occupational group from a geographical location with a low COVID-19 prevalence rate revealed no detectable SARS-CoV-2 infection. Further, there were limited COVID-19 cases within the hospital during the study, meaning that the risk of a health care professional learner contracting COVID-19 from a patient was low. RT-PCR NP swabs are the gold standard for identifying COVID-19 infection; however, they are resource-intensive requiring physician supervision, clerical, nursing and technical staff. Moreover, this testing occurred at a time of extremely high demand on the laboratory services. Throughout the pandemic, it has been essential to allocate resources appropriately while adapting to variants of concern such as B.1.1.529 (omicron). As vaccines have become widely available in Canada, it is crucial to continually evaluate the use of non-pharmacological interventions such as RT-PCR, rapid antigen tests, symptom screening tools, contact tracing and masking to determine what interventions will keep health care professional learners’ and the community safe. Our data suggests that negligible asymptomatic infection occurred in this group during a time of mandatory masking, physical distancing and restrictions on gathering. While the negative results with the gold standard test were reassuring in the context of the events that were unfolding, NP- RT-PCR testing was very resource intense and this an important consideration for future decision making in this and future pandemics.

Limitations of this study include sampling bias, as health care professional students who participated in this study may have engaged in less risky behaviour than some of their peers that were not enrolled in this study. NP SARS-CoV-2 RT-PCR testing was mandatory for all medical students to engage with in-person learning requirements, but they did not have to participate in this study in order to obtain testing. RT-PCR NP testing was free and accessible to students through our research study and public health. Whereas the other Queen’s FHS’ programs did not require mandatory testing the asymptomatic infection of COVID-19 is not as thoroughly captured in these specific student populations.

Two other respiratory viruses, influenza A&B and HOPV, were also not identified. Infections with these viruses were at a historic low. This is likely due to the low rates of these viruses circulating in the general population as a result of increased public health safety measures such as increased hand-washing, physical distancing, mask use, decreased contacts and higher influenza vaccine coverage [23–26].

Future directions include evaluating the seropositivity of this group due to previous infection and vaccine-induced immunity, and mental health outcomes.

## Data Availability

Data is available upon request.

## Abbreviations

COVID-19: Novel Coronavirus Disease of 2019
FHS: Faculty of Health Sciences
KFL&A: Kingston, Frontenac, Lennox & Addington
KHSC: Kingston Health Sciences
NP: Nasopharyngeal
RT-PCR: Reverse transcriptions-polymerase chain reaction
SARS-CoV-2: Severe-acute-respiratory coronavirus-2
HOPV: Human orthopneumovirus
